# Cardiovascular disease therapeutics via engineered oral microbiota: Applications and perspective

**DOI:** 10.1002/imt2.197

**Published:** 2024-05-09

**Authors:** Wenyu Zhen, Zifei Wang, Qing Wang, Wansu Sun, Rui Wang, Wenhao Zhang, Yulong Zhang, Wengang Qin, Bang Li, Qingqing Wang, Biao Hong, Yicheng Yang, Jing Xu, Siyu Ma, Ming Da, Linfei Feng, Xiaodong Zang, Xuming Mo, Xiaoyu Sun, Mingyue Wu, Junji Xu, Jianguang Xu, Yuan Huang, Hengguo Zhang

**Affiliations:** ^1^ Key Laboratory of Oral Diseases Research of Anhui Province, College & Hospital of Stomatology Anhui Medical University Hefei China; ^2^ Department of Stomatology The First Affiliated Hospital of Anhui Medical University Hefei China; ^3^ The First Affiliated Hospital of USTC, Division of Life Sciences and Medicine University of Science and Technology of China Hefei China; ^4^ State Key Laboratory of Cardiovascular Disease, Fuwai Hospital, National Center for Cardiovascular Diseases, Pediatric Cardiac Surgery Center, Fuwai Hospital Chinese Academy of Medical Sciences, and Peking Union Medical College Beijing China; ^5^ Department of Cardiothoracic Surgery Children's Hospital of Nanjing Medical University Nanjing China; ^6^ Laboratory of Tissue Regeneration and Immunology, Beijing Key Laboratory of Tooth Regeneration and Function Reconstruction, Department of Periodontics, School of Stomatology Capital Medical University Beijing China

**Keywords:** cardiovascular diseases, engineered microbiota, oral microbiota

## Abstract

Engineering bacteria are considered as a potential treatment for cardiovascular diseases and related risk factors. Oral bacteria are closely related to the occurrence and development of cardiovascular diseases, and their engineering has broad prospects and potential in the treatment of cardiovascular diseases. Oral pathogenic bacteria undergo protein and genetic engineering, including the incorporation of exogenous plasmids to yield therapeutic effects; genetically engineered oral probiotics can be harnessed to secrete cytokines and reactive oxygen species, offering novel therapeutic avenues for cardiovascular diseases.

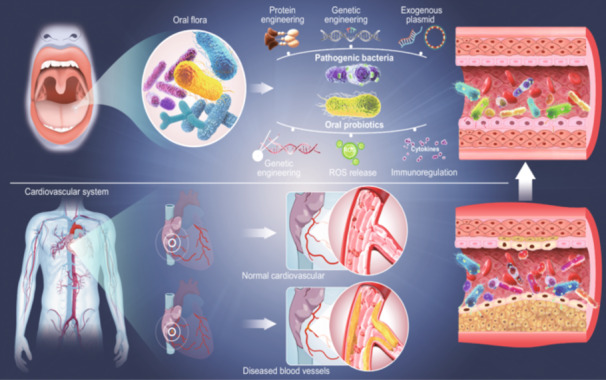

As the predominant cause of death worldwide, cardiovascular diseases (CVDs) are closely associated with systemic metabolic related obesity, hyperlipidemia, diabetes, and inflammatory bowel disease (IBD) [1]. In which, the microbiome manifests a critical role in the onset and progression of CVDs [2]. Consequently, microbiome‐based therapies, including antibiotics, probiotics, and fecal microbiota transplantation, have been increasingly utilized in CVDs management. Nevertheless, challenges such as resistance, precision, and safety of these therapies remain to be addressed. With the advancement of biosynthetic technology and a deeper understanding of the microbe‐host connection, there has been a shift toward engineering bacteria to surmount these hurdles [3].

The oral microbiota constitutes the second largest microbiome habitat in the human body. To date, approximately 700 species of oral bacteria have been identified, with 296 species classifiable within the oral cavity of an average person, collectively known as the human oral microbiota [4]. The dynamic imbalance between oral microbiota and the host is closely associated with the onset or progression of a range of systemic diseases [5]. A growing number of clinical surveys and epidemiological studies conducted in the past few years have demonstrated that oral dysbiosis can contribute to the development of CVDs [6]. Due to the vast and diverse oral microbiota, the engineered oral microbiota for CVDs treatment become novel and important. In light of this, we aim to explore the application of engineered microbiota in treating CVDs, and discuss the potential of engineered oral microbiota, grounded on research into the roles and mechanisms of oral microbiota in relation to CVDs.

## ASSOCIATION BETWEEN ORAL MICROBIOTA AND CARDIOVASCULAR DISEASES

The oral microbiota plays a crucial role in maintaining microbial homeostasis within the oral environment. Disruption of this balance, known as oral microbial dysbiosis, can lead to the onset of systemic diseases [5]. Periodontitis is a chronic inflammation of the periodontal supporting tissue caused by local factors. The main contributors to periodontal disease are bacteria presenting in dental plaque. *Porphyromonas gingivalis*, as a dominant bacterium of periodontitis, can stimulate the formation of foam cells, vascular smooth muscle cells proliferation and calcification, an increase in oxidative stress and inflammatory response in aortic endothelial cells [7]. It can also circulate with blood and lymph, avoid host immune clearance, produce local vascular inflammation, induce the production of autoimmune antibodies and systemic inflammatory mediators, and hasten the development of atherosclerosis [8]. In atherosclerotic plaques of myocardial infarction patients, *Aggregatibacter actinomycetemcomitans*, a bacterium causing periodontitis, was detected [9]. In addition, other oral pathogens are associated with CVDs, such as *Fusobacterium nucleatum*, which can mediate periodontitis to aggravate atherosclerosis by promoting hepatic glycolysis and adipogenesis [10]. Apart from causing bacteremia and infectious endocarditis, *Streptococcus mutans* is also seen in atherosclerotic plaques [11]. Research has indicated that a connection between hypertension and periodontitis, and a potential mechanism oral microbiota affecting blood pressure is through the inflammatory response linked to microbial homeostasis in periodontitis [12]. Furthermore, there exists a positive correlation between the surface area of periodontal inflammation and atrial fibrosis, suggesting that the former could serve as a risk factor for the latter [13].

## APPLICATION OF ENGINEERED MICROBIOTA IN CARDIOVASCULAR DISEASES

**Figure 1:**
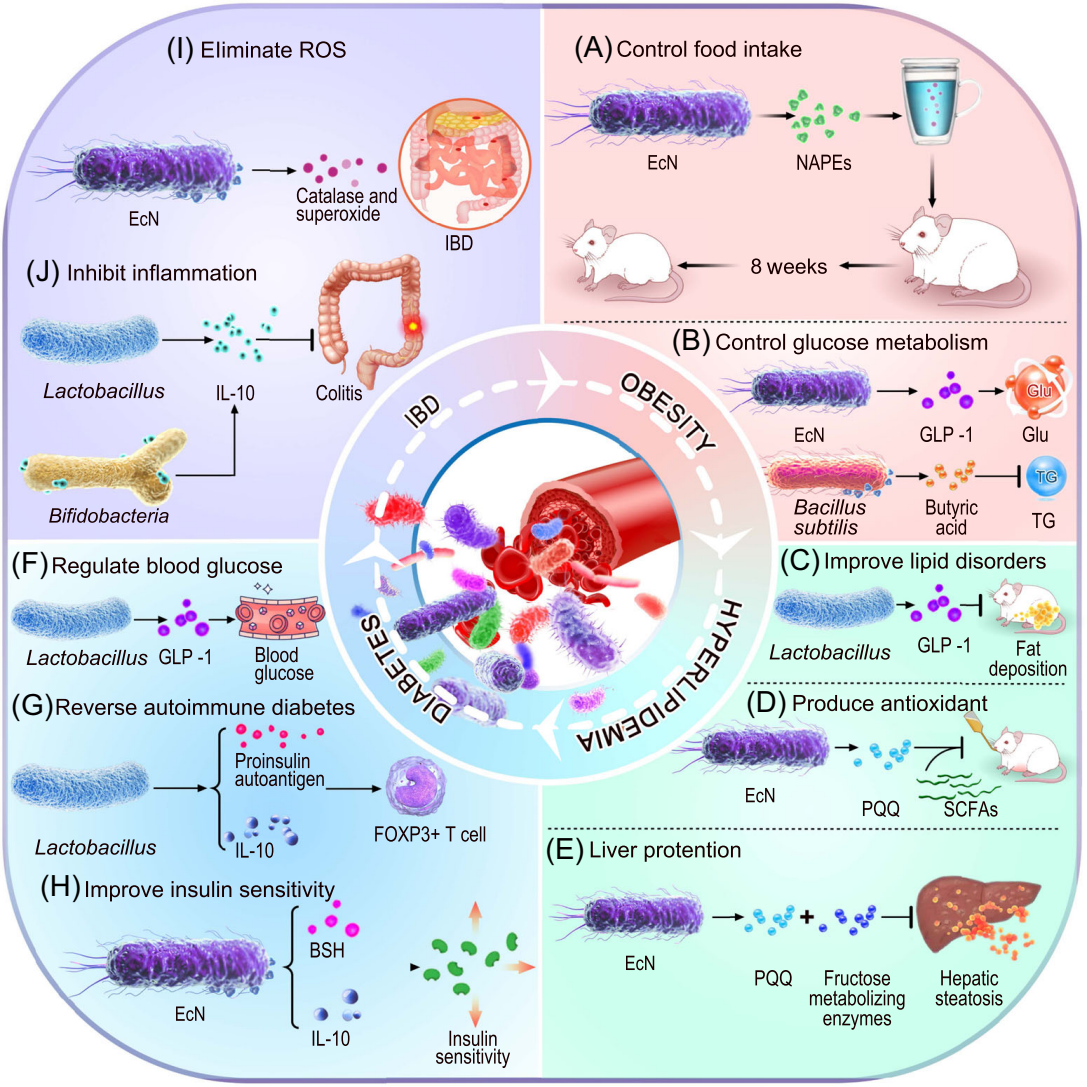
Application of engineering bacteria in cardiovascular diseases related risk factors. (A and B) Obesity: Engineered bacteria regulate food intake and glucose metabolism to fight obesity. (C–E) Hyperlipidemia: Engineering bacteria to produce glucagon‐like peptide‐1 (GLP‐1) and antioxidants enhance lipid metabolism, mitigate liver oxidative stress, and reduce hyperlipidemia. (F–H) Diabetes: Genetically engineered bacteria regulate blood glucose, reverse autoimmune diabetes, and enhance insulin sensitivity. (I and J) Inflammatory bowel disease (IBD): Engineered bacteria with enhanced antioxidant or anti‐inflammatory properties can treat IBD. BSH, bile salt hydrolase; EcN, *Escherichia coli* Nissle 1917; Glu, glucose; NAPEs, N‐acylphosphatidylethanolamines; PQQ, pyrroloquinoline quinone; SCFAs, short‐chain fatty acids; TG, triglyceride.

The onset of CVDs is often attributed to the multiple risk factors, such as obesity, hyperlipidemia, diabetes, and IBD. Engineered bacteria can effectively delay or prevent the occurrence of cardiovascular events (Figure 1). For obesity, genetically engineered bacteria expressing N‐acylphosphatidylethanolamines exhibit resistance to obesity caused by high‐fat diets (Figure 1A). Besides, modifying bacteria to secrete antioxidant can prevent liver oxidative damage and lessen hyperlipidemia (Figure 1D,E). It's feasible to utilize genetically modified bacteria with antioxidant enzymes or anti‐inflammatory cytokines for treating IBD (Figure 1I,J). Glucagon‐like peptide‐1 (GLP‐1) has the effect of regulating glucose metabolism and body weight, and can reduce liver fat deposition. Engineering bacteria secreting GLP‐1, such as *Escherichia coli* and *Lactococcus lactis*, can be built to play the above role (Figure 1B,C,F). GLP‐1 also has the function of reducing blood pressure. For example, the use of recombinant plasmids encoded by *hGLP* genes to transform *Clostridium butyricum* that produce butyric acid was shown to markedly improve myocardial hypertrophy and lower blood pressure, adjust microbiota imbalance, promote cardiac indicators, and activate AMPK/mTOR/p70S6K/4EBP1 signaling pathway in spontaneous hypertensive rats [14]. In which, synthetic biology provides tools for precisely controlling interactions between bacteria and host cells, enhancing the effectiveness and safety of bacterial therapies [15]. Bacteria's suitability for genetic modifications allows for the introduction of desirable traits, such as specific metabolic activities or production of beneficial metabolites, offering a novel approach to CVDs treatment.

## THE APPLICATION OF ORAL ENGINEERING MICROBIOTA IN CARDIOVASCULAR DISEASES

**Figure 2:**
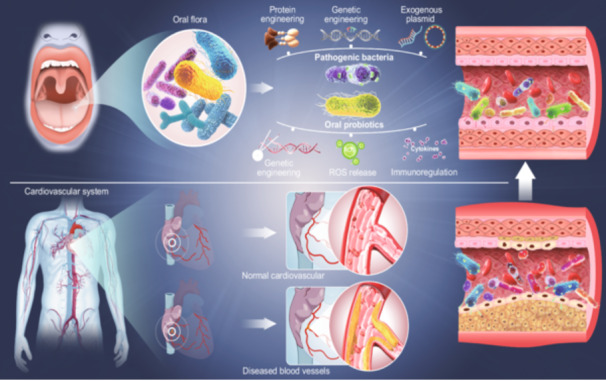
Schematic representation of the application of engineered bacteria in cardiovascular health. Oral pathogenic bacteria undergo protein and genetic engineering, including the incorporation of exogenous plasmids to yield therapeutic effects; genetically engineered oral probiotics can be harnessed to secrete cytokines and reactive oxygen species, offering novel therapeutic avenues for cardiovascular diseases.

As the engineered gut microbes effectively prevent and treat CVDs, the engineering and application of CVDs related oral microbes can potentially be regarded as the dawn of CVDs therapeutic. Importantly, the different engineering of oral probiotics and pathogenic bacteria provides multiple supports for specific and accurate CVDs therapies (Figure 2). Oral microbiota can be engineered by incorporating functional genes into plasmid vectors, transferring them into bacterial chassis, or directly integrating them into bacterial chromosomes. Engineered bacteria can deliver enzymes, anti‐inflammatory cytokines, and other bioactive molecules, precisely and effectively improving the accuracy and effectiveness of targeted interventions in the host microbiota [3].

The original probiotics can be engineered by gene editing tools to have beneficial characteristics, offering targeted treatments for specific diseases [15]. Certain *Lactobacillus* strains, such as *Lactobacillus fermentans, Lactobacillus rhamnosus, Lactobacillus plantarum*, and *Lactobacillus casei*, are among the potential candidates for oral probiotics. These strains are nonpathogenic and demonstrate promising probiotic potential [16]. Engineered *L. plantarum* has been used in cardiovascular related treatments. For instance, recombinant *L. plantarum* can regulate diabetes and hypertension by secreting angiotensin (1−7). Moreover, excessive accumulation of reactive oxygen species can cause oxidative stress, which is characterized by an imbalance between oxidation and antioxidant actions and plays a significant role in the onset and progression of CVDs. Remarkably, the recombinant *L. plantarum* NC8‐pSIP409‐alr‐ACEIP strain has been shown to protect human umbilical vein endothelial cells against hydrogen peroxide‐induced oxidative stress, reducing hypertension‐related angiotensin II protein levels while enhancing angiotensin‐converting enzyme 2 protein expression in these cells [17]. In addition, engineered oral probiotics can also exhibit anti‐inflammatory properties. Engineering *L. lactis* to express the LZ8 protein has shown promise in mitigating early atherosclerosis. This innovative approach has been demonstrated to suppress the gene expression of the IL‐1β in the aorta, reduce the thickness of the intima‐media and the presence of subendothelial foam cells, thereby exerting anti‐inflammatory effects. Such advancements position this engineered bacterium as a potential therapeutic agent for improving early stages of atherosclerosis [18] (Figure 2).

In addition to oral probiotics, oral pathogens associated with CVDs can be modified through protein engineering, genetic engineering, exogenous plasmid, and other methods (Figure 2). For example, researchers have devised a self‐targeting CRISPR array, which, when introduced into *S. mutans* UA159, effectively edits *gtfB* or *gtfB/gtfC* genes, resulting in a significant reduction in the synthesis of extracellular polysaccharides and the inhibition of biofilm formation [19]. The strong cariogenicity of *S. mutans* mainly depends on its acidogenicity and acid tolerance. Since the bacteria are usually delivered directly through oral administration, they need to be protected from the harsh environment of the oral cavity and gastrointestinal tract. Because of its high acid tolerance, *S. mutans* can survive and even manufacture acid at pH 4.5, which is better for preserving its viability and ability to proliferate once it reaches the target location.

## PROSPECTS

Based on the applications of engineered gut microbiota in CVDs treatment and the participation of oral microbiota in CVDs occurrence or progression, the engineering of oral microbiota possesses broad challenges and prospects. First, as the second largest microbial community in the human body, the oral microbiota contains different morphologies, substrates, and stability in different parts. This diversity provides a rich array of microhabitats, making the oral microbiome a versatile target for microbial engineering. Second, the oral cavity serves as the entry point to the digestive and respiratory systems, positioning it as a crucial interface between the internal body and the external environment [4]. More importantly, the collection methods of oral microbial samples are more convenient and diverse, making it easier for patients to accept. Moreover, appropriate sampling methods can be selected according to research purposes, which is conducive to bacterial engineering [20].

In addition, focal infections originating from the oral cavity, such as those found in periodontal pockets and extraction sockets, present a pathway for microbiota to invade deep tissues and contribute to systemic inflammation. These microorganisms can disseminate through various routes, including connective tissues, muscle and fascial planes, bone cavities, blood or lymphatic vessels, nerves, or across the salivary gland mucosa surface. Such dissemination is closely linked to systemic inflammatory responses, thereby expanding the potential targets for colonization by engineered oral microbiota [4]. The investigation into the intricate relationship and mechanisms underlying the interaction between oral microbiota and CVDs lays a robust theoretical foundation for the precision engineering of oral microbiota as a targeted intervention strategy in CVDs treatment. Therefore, we can target engineering oral bacteria to achieve personalized treatment by analyzing patient specific factors like genetic background, such as *P. gingivalis*. It is noteworthy that several engineered probiotics have entered the clinical trial stage, such as genetically engineered *L. lactis* AG019 for the treatment of type I diabetes, which allows us to see the possibility of oral microbial engineering in clinical application.

Despite the undeniable advantages of utilizing engineered bacteria in disease management, several challenges and limitations persist. The modified bacteria still belong to live bacteria, and their bacterial virulence and genetic instability may provoke adverse reactions or even harmful effects in the host. The risk can be mitigated by knocking out or mutating virulence‐related genes, regulating the number of bacteria, and screening non‐pathogenic bacteria. However, engineered oral microbiota are still in the early stages, and more stable synthetic tools still need to be developed to address safety concerns. Furthermore, the therapeutic use of bacteria‐related microbes needs to follow the regulations and guidelines of regulatory authorities to ensure their efficacy and safety. Additionally, the efficiency of utilization, colonization, and transformation of engineered bacteria needs further enhancement. For the sake of environmental health, cost savings, improved production efficiency, and the development of greener and more sustainable bioactive materials is something we need to pay attention to. Moreover, the clinical diversity of CVDs phenotypes complicates the accurate description and understanding of these conditions, posing a barrier to effective communication between clinicians and researchers. Addressing these challenges requires not only advancing our scientific understanding and technological capabilities but also refining the clinical categorization and characterization of CVDs to facilitate more effective interventions.

Despite these hurdles, the potential of engineered oral microbiota in mitigating CVDs risks highlights a promising area of research. Gene recombination technology is growing more capable, and there is a continuous development of new and low cost‐effective tools for large‐scale genetic operations. Continued exploration and innovation in this field could lead to breakthroughs in preventing and treating CVDs through targeted microbial engineering.

## AUTHOR CONTRIBUTIONS

Hengguo Zhang, Yuan Huang, and Jianguang Xu provided direction and guidance throughout the preparation of this manuscript. Wenyu Zhen, Zifei Wang, and Wansu Sun wrote and edited the manuscript. Zifei Wang, Rui Wang, Wengang Qin, Wenhao Zhang, Bang Li, Qingqing Wang, Biao Hong, Xiaoyu Sun, and Mingyue Wu generated and revised the figures. Wenyu Zhen, Yulong Zhang, Qing Wang, Yicheng Yang, Jing Xu, Siyu Ma, Ming Da, Linfei Feng, Xiaodong Zang, Xuming Mo, and Junji Xu revised the manuscript. All authors have read and approved the final manuscript.

## CONFLICT OF INTEREST STATEMENT

The authors declare no conflict of interest.

## ETHICS STATEMENT

No animals or humans were involved in this study.

## Data Availability

This manuscript does not generate any code or data. Supplementary materials (graphical abstract, slides, videos, Chinese translated version) may be found in the online DOI or iMeta Science http://www.imeta.science/.
